# Association of endovascular treatment versus intravenous thrombolysis in mild stroke across occlusion subgroups

**DOI:** 10.3389/fneur.2026.1878069

**Published:** 2026-10-14

**Authors:** Gao-Peng Xing, Xiao-Long Li, Ting-Ting Wang, Hui-Sheng Chen

**Affiliations:** 1 Department of Neurology, General Hospital of Northern Theater Command, Shenyang, China; 2 Department of Graduate School, Dalian Medical University, Dalian, China

**Keywords:** endovascular treatment, large vessel occlusion, minor stroke, occlusion site, outcome

## Abstract

**Background:**

The benefit of endovascular treatment (EVT) vs. intravenous thrombolysis (IVT) is uncertain in minor stroke patients (National Institutes of Health Stroke Scale [NIHSS] ≤ 5) with anterior large vessel occlusion (LVO).

**Methods:**

The pooled cohort consisted of patients with NIHSS ≤ 5 and internal carotid artery (ICA), M1 or M2 segment of the middle cerebral artery (MCA) from the DETECT2-China study and the Early antiplatelet for minor stroke following thrombolysis (EAST) trial. The primary efficacy outcome was excellent functional outcome, defined as a mRS 0–1 at 90 days. The primary safety outcome was symptomatic intracranial hemorrhage (sICH). Subgroup analyses were performed using interaction terms in logistic regression models to assess effect modification by baseline variable. To further explore the subgroup findings, analyses were repeated separately in patients with ICA/MCA-M1 occlusion and those with MCA-M2 occlusion.

**Results:**

A total of 237 patients were enrolled, including 105 (44.3%) with the IVT group, 132 (55.7%) with the EVT group. Compared to the IVT group, the EVT group had a similar proportion of excellent functional outcome (73.5% vs. 75.2%, aOR = 0.749, 95% CI = 0.392–1.433, *p* = 0.383), a numerically higher proportion of sICH (1.9% vs. 6.1%, aOR = 4.158, 95% CI = 0.819–21.127, *p* = 0.086). Subgroup analysis revealed that a numerically increased odds of excellent functional outcome was found in patients with ICA or MCA-M1 occlusion who received EVT vs. IVT (79.4% vs. 67.2%, aOR = 1.491, 95% CI: 0.693–3.211; *p* = 0.307), whereas significantly less probability of excellent functional outcome in those with MCA-M2 occlusion who received EVT vs. IVT (53.3% vs. 86.4%, aOR = 0.250, 95% CI: 0.057–0.980, *p* = 0.050). A significant interaction effect was identified between treatment (EVT vs. IVT) and excellent functional outcome (*p* for interaction = 0.008).

**Conclusion:**

In this exploratory analysis, the association between reperfusion strategy and functional outcome differed according to occlusion site. Patients with proximal occlusions (ICA or MCA-M1) in the EVT cohort had numerically better outcomes, whereas those with M2 occlusions in the EVT cohort had worse outcomes compared with the IVT cohort.

## Introduction

Minor stroke, defined as an acute ischemic stroke (AIS) with a National Institutes of Health Stroke Scale (NIHSS) score ≤ 5, accounts for approximately half of all AIS cases (1–3). Among these patients, 4 to 12% have large vessel occlusion (LVO) (4, 5). Although intravenous thrombolysis (IVT) is standard care for minor disabling stroke with LVO, a considerable proportion of these patients still experience neurological deterioration and poor functional outcomes (4–8). However, the benefit of endovascular therapy (EVT) remains uncertain in this population, as the randomized clinical trials (RCTs) that established the efficacy and safety of EVT in LVO largely excluded patients with NIHSS ≤ 5 (9–15). Consequently, while current international guidelines strongly recommend EVT for AIS patients with LVO and NIHSS ≥ 6 (16), there are no established recommendations for those with NIHSS scores of 0–5.

Several observational studies have evaluated the efficacy and safety of endovascular therapy (EVT) in mild stroke patients with large vessel occlusion (LVO) (17–21), yet their findings remain inconsistent. While EVT did not significantly improve clinical outcomes and was associated with an increased risk of symptomatic intracranial hemorrhage (sICH) in one study (17), others suggested potential benefits for those with proximal vessel occlusions (18). Similarly, prior research has shown that despite treatment with intravenous thrombolysis (IVT), patients with proximal LVO [such as in the internal carotid artery (ICA) or M1 segment of the middle cerebral artery (MCA)] face a significantly higher risk of early neurological deterioration, whereas those with M2 occlusions may derive greater benefit from IVT (8).

The EVT in patients presenting with low NIHSS scores (≤5) is increasingly regarded as a potential frontier in endovascular stroke treatment. Two ongoing randomized trials—Endovascular Therapy for Low NIHSS Ischemic Strokes (ENDOLOW) and Minor Stroke Therapy Evaluation (MOSTE)—are currently investigating the efficacy of EVT in AIS due to LVO and NIHSS ≤ 5, focusing primarily on proximal ICA or M1 occlusions. The results of these trials are expected to clarify whether EVT provides meaningful benefits in mild ischemic strokes with proximal ICA or M1 occlusions (22, 23).

Given the current evidence, the benefit of EVT versus IVT in minor stroke patients with LVO remains uncertain. In this context, we aimed to evaluate the effectiveness and safety of EVT compared to IVT in minor stroke patients with anterior circulation LVO and to identify specific subgroups that may benefit from EVT.

## Methods

### Patient selection

From two independent cohorts, we screened consecutive patients with AIS-LVO from May 2018 to December 2024. DETECT2-China (Endovascular Treatment for Acute Ischemic Stroke in China, NCT05092139) is a prospective national multicenter registry study, evaluating AIS patients treated with EVT in China. The design and methodology of the registry have been described in detail in the published protocol (24). The EAST (Early Antiplatelet for Minor Stroke following Thrombolysis, NCT05193071) is a prospective, double-blind, multicenter, randomized, placebo-controlled study conducted in China, which aimed to evaluate the efficacy and safety of early antiplatelet therapy initiated within 6 h after IVT in minor stroke patients. Details of the study design and rationale have been published (25).

Inclusion criteria were as follows: (1) age ≥ 18 years; (2) pre-stroke modified Rankin Scale (mRS) score ≤ 1; (3) acute anterior circulation LVO; (4) minor stroke presentation, defined as a baseline NIHSS score ≤ 5; and (5) availability of complete 90-day mRS follow-up data. Patients were excluded if they had missing procedural details, a pre-stroke mRS score ≥2, insufficient neuroimaging data, or underwent rescue EVT after intravenous thrombolysis. Written informed consent was obtained from all patients or their legal representatives. Both studies were approved by the institutional review board of the General Hospital of Northern Theater Command and adhered to the ethical standards outlined in the Declaration of Helsinki.

### Study treatments, exposures, and interventions

Based on the treatment regimen, eligible patients were categorized into two groups: the EVT group, which underwent endovascular therapy, and the IVT group, which received intravenous thrombolysis. EVT was performed using a stent retriever, an aspiration device, or a combination of both techniques. The use of intra-arterial thrombolytics was left to the discretion of the neurointerventionalist.

### Data collection

Demographic characteristics, history of cerebrovascular disease, clinical presentation, imaging findings, and procedural details were prospectively collected. Functional outcomes were assessed using mRS at 90 days by certified neurologists during telephone interviews or in-person visits.

### Clinical outcomes

The primary efficacy outcome was an excellent functional outcome, defined as an mRS score of 0 or 1 at 90 days. Secondary efficacy outcomes included a favorable functional outcome (mRS 0–2 at 90 days) and the distribution of disability scores across the mRS. Safety outcomes comprised symptomatic intracranial hemorrhage (sICH), characterized as an increase of ≥4 points on the NIHSS within 24 h attributed to intracranial hemorrhage, and early neurological deterioration (END), defined as an increase of ≥4 points on the NIHSS within 24 h.

### Statistical analysis

Quantitative variables were expressed as mean ± standard deviation (SD) for normally distributed data or median with interquartile range (IQR) for skewed distributions. Group differences in quantitative variables were analyzed using Student’s *t*-test (for normally distributed parameters) or the Mann–Whitney *U* test (for non-normally distributed parameters). Categorical variables were summarized as numbers (percentages) and compared using the *χ*^2^ test or Fisher’s exact test, as appropriate.

Binary logistic regression was performed to assess clinical outcomes, including excellent and favorable functional outcomes at 90 days, END, and sICH. Results are reported as odds ratios (OR) with 95% confidence intervals (CI). An ordinal logistic regression model was used to perform shift analysis on the 90-day mRS scores across treatment groups. Multivariable models were adjusted using a consistent set of clinically relevant covariates selected *a priori* based on clinical relevance and previous literature. For the overall cohort, the model included age, baseline NIHSS score, prestroke mRS score, and occlusion site. In analyses restricted to a specific occlusion-site subgroup, occlusion site was omitted because it had no within-subgroup variation, while the remaining covariates were retained. Covariates were not selected according to univariable *p*-values. Analyses with insufficient outcome events were reported descriptively.

Subgroup analyses were conducted across clinically relevant factors, including age, sex, baseline NIHSS score, baseline systolic blood pressure (SBP), presumed stroke etiology, and occluded vessel site. Effect modification was assessed by introducing interaction terms between each subgroup variable and treatment assignment into multivariable logistic regression models, with significance evaluated using the Wald test. Stratified analyses within each subgroup were performed to estimate subgroup-specific ORs and 95% CIs.

To evaluate the robustness of the primary findings, sensitivity analyses were carried out by repeating the main analyses in two predefined populations: patients with proximal vessel occlusions (ICA or MCA M1) and those with MCA M2 occlusion. Given the limited sample size and sparse data in the MCA M2 subgroup, Fisher’s exact test and Firth’s regression were used for all between-group comparisons of clinical outcomes. No formal adjustment for multiple comparisons was performed. Accordingly, *p*-values for secondary outcomes, subgroup analyses, and interaction tests are nominal and should be interpreted as exploratory and hypothesis-generating. All analyses were performed using R software.

## Results

### Baseline characteristics

A total of 237 patients were enrolled, including 105 (44.3%) in the IVT group, 132 (55.7%) in the EVT group (Figure 1). Among the 132 patients in the EVT cohort, 16 (12.1%) received bridging therapy (IVT followed by EVT), whereas the remaining 116 (87.9%) underwent direct EVT. Compared with the IVT group, the patients with EVT have more drinkers (41.7% vs. 23.8%, *p* = 0.004) and a higher proximal vessel occlusion site (ICA or MCA-M1) (77.3% vs. 58.1%, *p* = 0.002) and less rate of past medical history of coronary heart disease (2.3% vs. 7.6%, *p* = 0.052; Table 1).

**Figure 1:**
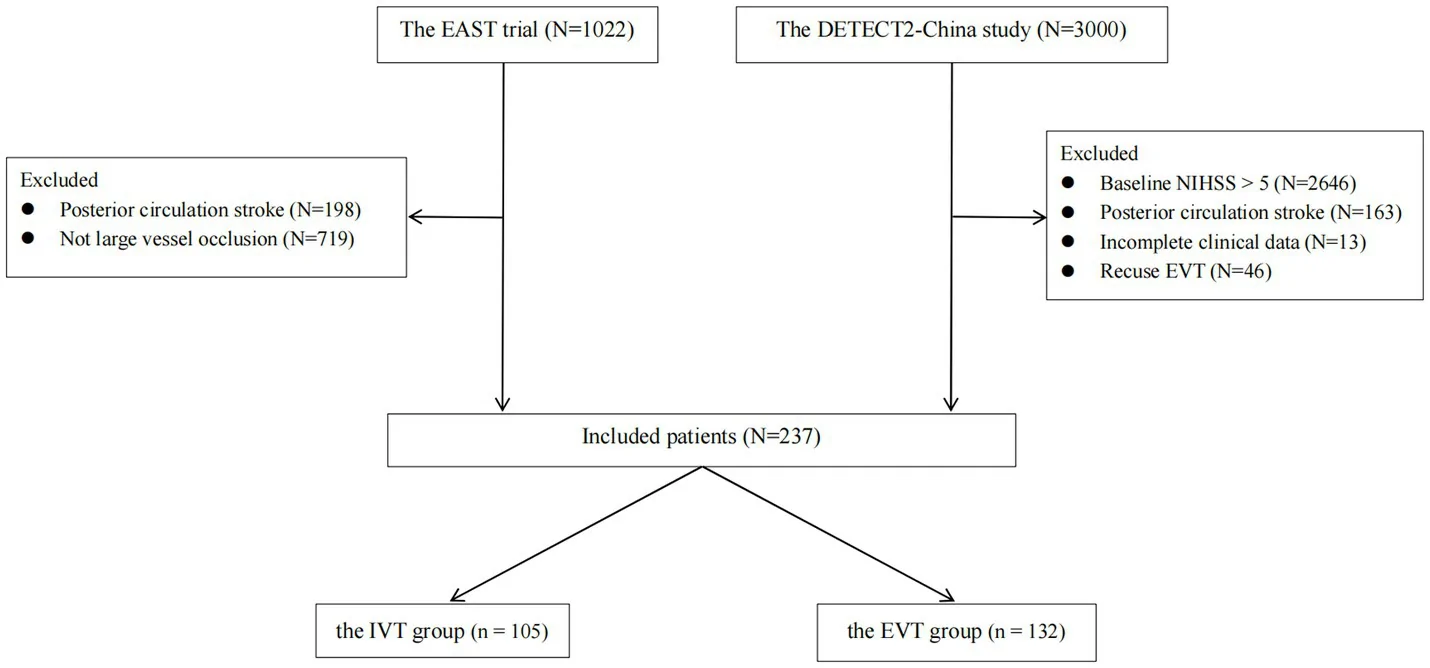
Flowchart of the subject selection. EVT, endovascular treatment; IVT, intravenous thrombolysis.

**Table 1:** Baseline characteristics of enrolled patients.

Variable	Overall (*n* = 237)	IVT (*n* = 105)	EVT (*n* = 132)	*p*
Age – year	66.0 (60.0–71.0)	67.0 (61.0–73.0)	66.0 (59.8–70.0)	0.124
Male	191 (80.6)	85 (81.0)	106 (80.3)	0.900
Current Smoking	106 (44.7)	44 (41.9)	62 (47.0)	0.436
Current Drinking	80 (33.8)	25 (23.8)	55 (41.7)	0.004
Past medical history				
Hypertension	142 (59.9)	64 (61.0)	78 (59.1)	0.771
Diabetes	57 (24.1)	25 (23.8)	32 (24.2)	0.938
Coronary heart disease	11 (4.6)	8 (7.6)	3 (2.3)	0.052
Atrial fibrillation	13 (5.5)	6 (5.7)	7 (5.3)	0.890
Ischemic stroke	53 (22.4)	26 (24.8)	27 (20.5)	0.429
Baseline NIHSS	4.0 (2.0–5.0)	3.0 (2.0–5.0)	4.0 (3.0–5.0)	0.234
SBP at admission	144.0 (130.5–158.2)	143.5 (131.8–158.0)	144.0 (128.0–161.0)	0.964
DBP at admission	82.0 (75.0–90.0)	82.0 (75.0–89.2)	82.0 (75.0–90.5)	0.817
Prestroke mRS	0.0 (0.0–0.0)	0.0 (0.0–0.0)	0.0 (0.0–0.0)	0.682
Presumed stroke cause^a^				0.948
Large artery atherosclerosis	217 (91.6)	96 (91.4)	121 (91.7)	
Cardioembolism	20 (8.3)	9 (8.6)	11 (8.3)	
Responsible vessel occlusion site				0.002
ICA or MCA-M1	163 (68.8)	61 (58.1)	102 (77.3)	
MCA-M2	74 (31.2)	44 (41.9)	30 (22.7)	

Data were shown as *N* (%) or median (IQR). NIHSS, National Institutes of Health Stroke Scale; SBP, systolic blood pressure; DBP, diastolic blood pressure; IVT, intravenous thrombolysis; EVT, endovascular treatment; mRS, modified Rankin Scale; ICA, internal carotid artery; MCA, middle cerebral artery; MCA-M1, M1 segment of middle cerebral artery; MCA-M2, M2 segment of middle cerebral artery. ^a^The presumed stroke cause was classified according to the Trial of Org 10,172 in Acute Stroke Treatment (TOAST) classification system.

### Clinical outcomes

No significant difference was observed between the IVT and EVT groups in terms of excellent functional outcome (73.5% vs. 75.2%, aOR = 0.749, 95% CI = 0.392–1.433, *p* = 0.383) or favorable functional outcome (82.6% vs. 84.8%, aOR = 0.673, 95% CI = 0.317–1.426, *p* = 0.301). However, a numerically higher incidence of sICH was observed in the EVT group compared to the IVT group (6.1% vs. 1.9%, aOR = 4.158, 95% CI = 0.819–21.127, *p* = 0.086). Additionally, the EVT group exhibited a significantly higher rate of END (14.5% vs. 3.8%, aOR = 5.046, 95% CI = 1.591–16.002, *p* = 0.006; Table 2 and Figure 2A). Unadjusted clinical outcomes stratified by treatment strategy are presented in Table 3.

**Table 2:** Efficacy and safety outcomes.

Outcome	IVT (*n* = 105)	EVT (*n* = 132)	Unadjusted OR (95% CI)	*P*	Adjusted OR (95% CI)^a^	*P* ^*^
Excellent functional outcome	79 (75.2)	97 (73.5)	0.912 (0.507–1.642)	0.759	0.749 (0.392–1.433)	0.383
Favorable functional outcome	89 (84.8)	109 (82.6)	0.852 (0.424–1.710)	0.652	0.673 (0.317–1.426)	0.301
mRS distribution at 90 days^b^	0.0 (0.0–1.0)	1.0 (0.0–2.0)	2.009 (1.229–3.285)	0.005	2.177 (1.290–3.676)	0.004
Early neurological deterioration	4 (3.8)	19 (14.5)	3.910 (1.469–12.845)	0.005	5.046 (1.591–16.002)	0.006
Any intracranial hemorrhage	3 (2.9)	21 (15.9)	5.647 (1.984–21.692)	<0.001	10.135 (2.269–45.265)	0.002
Symptomatic intracranial hemorrhage	2 (1.9)	8 (6.1)	2.827 (0.760–15.185)	0.126	4.158 (0.819–21.127)	0.086

Data were shown as *N* (%) or median (IQR). OR, odds ratio; CI, confidence interval; mRS, modified Rankin Scale. ^a^Adjusted for the variables with age, baseline NIHSS score, prestroke mRS score and responsible vessel occlusion site. ^b^mRS scores range from 0 to 6: 0 = no symptoms; 1 = symptoms without clinically significant disability; 2 = slight disability; 3 = moderate disability; 4 = moderately severe disability; 5 = severe disability; and 6 = death. *Firth’s penalized logistic regression was used for binary outcomes with sparse events.

**Figure 2:**
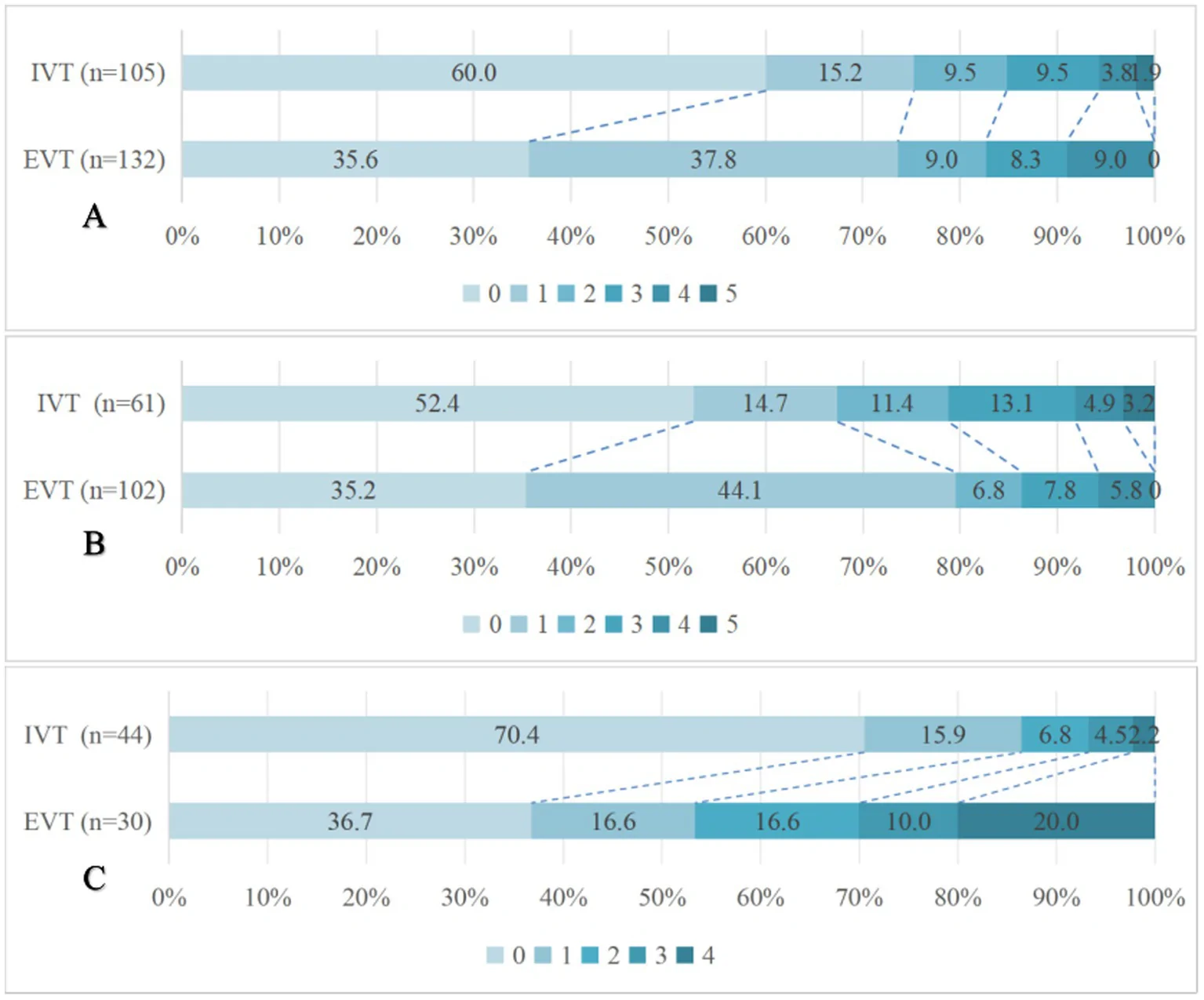
Distribution of modified Rankin Scale (mRS) scores at 90 days. The distributions of 90-day mRS scores are shown for **(A)** the overall study population, **(B)** patients with proximal vessel occlusion (ICA or MCA-M1), and **(C)** patients with MCA-M2 occlusion. Scores range from 0 to 6 (0 = no symptoms, 1 = symptoms without clinically significant disability, 2 = slight disability, 3 = moderate disability, 4 = moderately severe disability, 5 = severe disability, and 6 = death). ICA, internal carotid artery; MCA-M1, M1 segment of middle cerebral artery; MCA-M2, M2 segment of middle cerebral artery; EVT, endovascular treatment; IVT, intravenous thrombolysis.

**Table 3:** Efficacy and safety outcomes according to treatment strategy.

Outcome	IVT only (*n* = 105)	direct EVT (*n* = 116)	Bridging Therapy (*n* = 16)	*P* ^*^
Excellent functional outcome	79 (75.2)	83 (71.6)	14 (87.5)	0.374
Favorable functional outcome	89 (84.8)	95 (81.9)	14 (87.5)	0.769
mRS distribution at 90 days^a^	0.0 (0.0–1.0)	1.0 (0.0–2.0)	0.0 (0.0–1.0)	0.003
Early neurological deterioration	4 (3.8)	16 (13.8)	3 (18.8)	0.020
Any intracranial hemorrhage	3 (2.9)	20 (17.2)	1 (6.3)	0.002
Symptomatic intracranial hemorrhage	2 (1.9)	8 (6.9)	0 (0.0)	0.164

Data were shown as *N* (%) or median (IQR). IVT, intravenous thrombolysis; EVT, endovascular treatment; mRS, modified Rankin Scale. ^a^mRS scores range from 0 to 6: 0 = no symptoms; 1 = symptoms without clinically significant disability; 2 = slight disability; 3 = moderate disability; 4 = moderately severe disability; 5 = severe disability; and 6 = death. *p-values were obtained using unadjusted comparisons (*χ*^2^ test, Fisher’s exact test, or Mann–Whitney *U* test, as appropriate). Because of the limited sample size and sparse outcome events, adjusted regression analyses were not performed, and the findings should be interpreted as exploratory.

In prespecified subgroup analyses, no statistically significant interaction was observed between treatment assignment and most subgroup variables, including age, sex, baseline NIHSS score, baseline systolic blood pressure, and presumed stroke etiology (Figure 3). However, a significant interaction was identified between treatment group and occlusion site in relation to excellent functional outcome (*P* for interaction = 0.008). Among patients with proximal vessel occlusion (ICA or MCA-M1), EVT was associated with a trend toward higher rates of excellent functional outcome compared to IVT (79.4% vs. 67.2%; aOR = 1.491, 95% CI: 0.693–3.211; *p* = 0.307). In contrast, among those with MCA-M2 occlusion, the EVT cohort had a lower proportion of excellent functional outcome compared with the IVT cohort (53.3% vs. 86.4%; aOR = 0.250, 95% CI: 0.057–0.980; *p* = 0.050; Figure 3).

**Figure 3:**
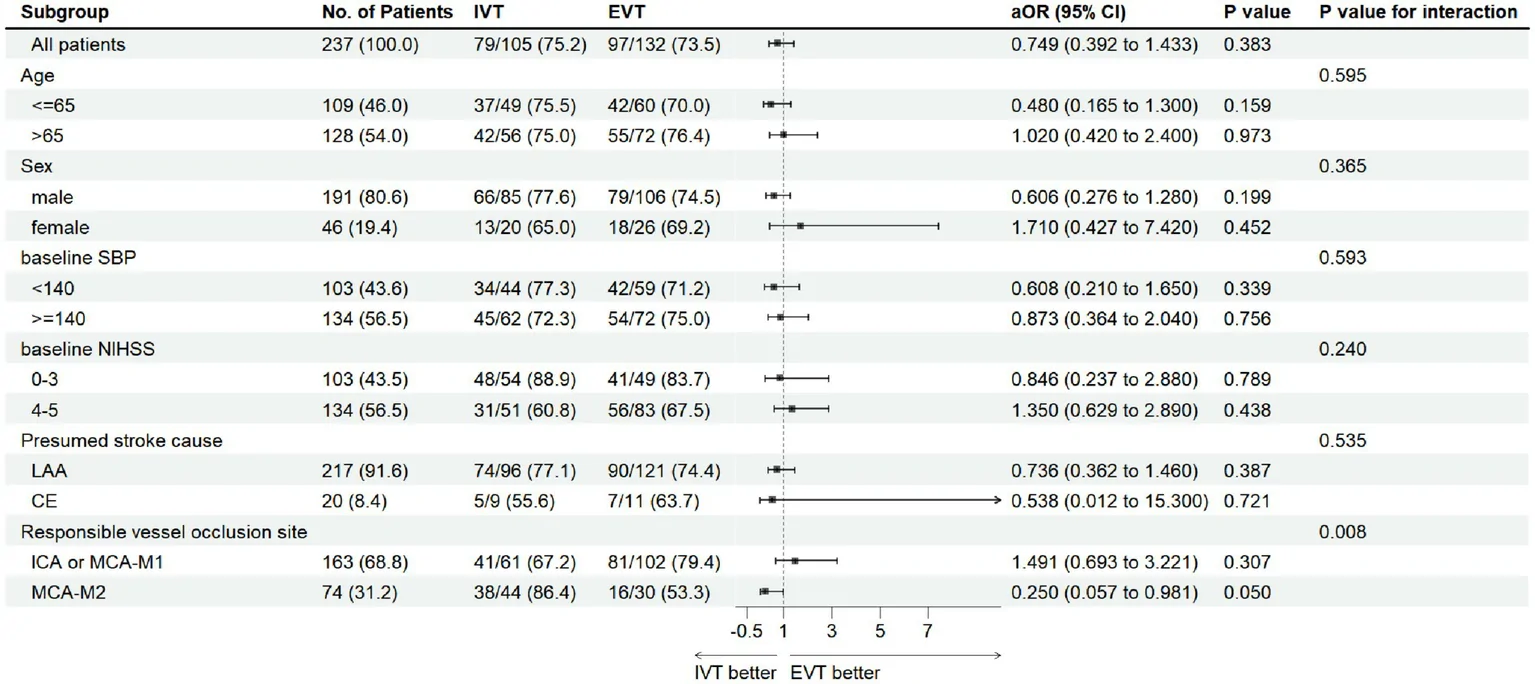
Primary outcome by subgroups. EVT, endovascular treatment; IVT, intravenous thrombolysis.

### Outcomes in patients with proximal vessel occlusion (ICA or MCA-M1)

A total of 163 patients had proximal vessel occlusion, including 61 (37.4%) in the IVT group and 102 (62.6%) in the EVT group (Table 4). Compared with the IVT group, the EVT group showed a numerically higher proportion of excellent functional outcome (79.4% vs. 67.2%; aOR = 1.491, 95% CI: 0.693–3.211; *p* = 0.307) and favorable functional outcome (86.3% vs. 78.7%; aOR = 1.443, 95% CI: 0.600–3.470; *p* = 0.413), as well as a lower median 90-day mRS score (0.0 [IQR 0.0–2.0] vs. 1.0 [IQR 0.0–1.0]; aOR = 1.445, 95% CI: 0.773–2.700; *p* = 0.249) (Table 5 and Figure 2B).

**Table 4:** Baseline characteristics of patients with proximal vessel occlusion (ICA or MCA-M1).

Variable	Overall (*n* = 163)	IVT (*n* = 61)	EVT (*n* = 102)	*p*
Age – year	67.0 (61.0–71.0)	68.0 (61.0–74.0)	67.0 (61.0–70.8)	0.123
Male	131 (80.4)	50 (82.0)	81 (79.4)	0.691
Current Smoking	79 (48.5)	31 (50.8)	48 (47.1)	0.642
Current Drinking	63 (38.7)	20 (32.8)	43 (42.2)	0.234
Past medical history				
Hypertension	100 (61.3)	35 (57.4)	65 (63.7)	0.421
Diabetes	45 (27.6)	17 (27.9)	28 (27.5)	0.954
Coronary heart disease	7 (4.3)	5 (8.2)	2 (2.0)	0.057
Atrial fibrillation	6 (3.7)	2 (3.3)	4 (3.9)	0.833
Ischemic stroke	27 (16.6)	10 (16.4)	17 (16.7)	0.964
Baseline NIHSS	4.0 (3.0–5.0)	4.0 (3.0–5.0)	4.0 (3.0–5.0)	0.185
SBP at admission	144.0 (129.2–158.4)	143.0 (130.0–158.0)	144.0 (128.0–159.0)	0.990
DBP at admission	82.0 (75.0–90.0)	80.0 (75.0–90.0)	82.0 (75.0–90.0)	0.765
Prestroke mRS	0.0 (0.0–0.0)	0.0 (0.0–0.0)	0.0 (0.0–0.0)	0.564
Presumed stroke cause^a^				0.309
Large artery atherosclerosis	149 (91.4)	54 (88.5)	95 (93.1)	
Cardioembolism	14 (8.6)	7 (11.5)	7 (6.9)	

Data were shown as *N* (%) or median (IQR). ICA, internal carotid artery; MCA-M1, M1 segment of middle cerebral artery; NIHSS, National Institutes of Health Stroke Scale; SBP, systolic blood pressure; DBP, diastolic blood pressure; IVT, intravenous thrombolysis; EVT, endovascular treatment; mRS, modified Rankin Scale. a The presumed stroke cause was classified according to the Trial of Org 10,172 in Acute Stroke Treatment (TOAST) classification system.

**Table 5:** Efficacy and safety outcomes of patients with proximal vessel occlusion (ICA or MCA-M1).

Outcome	IVT (*n* = 61)	EVT (*n* = 102)	Unadjusted OR (95% CI)	*P*	Adjusted OR (95% CI)^a^	*P* ^*^
Excellent functional outcome	41 (67.2)	81 (79.4)	1.882 (0.917–3.859)	0.084	1.491 (0.693–3.211)	0.307
Favorable functional outcome	48 (78.7)	88 (86.3)	1.702 (0.740–3.915)	0.211	1.443 (0.600–3.470)	0.413
mRS distribution at 90 days^b^	0.0 (0.0–2.0)	1.0 (0.0–1.0)	1.233 (0.673–2.257)	0.498	1.445 (0.773–2.700)	0.249
Early neurological deterioration	4 (6.6)	12 (11.8)	1.763 (0.543–5.725)	0.420	2.130 (0.618–7.344)	0.231
Any intracranial hemorrhage	2 (3.3)	15 (14.7)	4.216 (1.242–21.855)	0.019	5.027 (1.083–23.334)	0.039
Symptomatic intracranial hemorrhage	2 (3.3)	4 (3.9)	1.087 (0.233–6.394)	0.917	1.311 (0.217–7.938)	0.768

Data were shown as *N* (%) or median (IQR). ICA, internal carotid artery; MCA-M1, M1 segment of middle cerebral artery MCA; OR, odds ratio; CI, confidence interval; mRS, modified Rankin Scale. ^a^Adjusted for the variables with age, baseline NIHSS score and prestroke mRS score. ^b^mRS scores range from 0 to 6: 0 = no symptoms; 1 = symptoms without clinically significant disability; 2 = slight disability; 3 = moderate disability; 4 = moderately severe disability; 5 = severe disability; and 6 = death. *Firth’s penalized logistic regression was used for binary outcomes with sparse events.

### Outcomes in patients with MCA-M2 occlusion

A total of 74 patients had MCA-M2 occlusion, including 44 (59.5%) in the IVT cohort and 30 (40.5%) in the EVT cohort (Table 6). Excellent functional outcome was observed in 38 of 44 patients (86.4%) in the IVT cohort and 16 of 30 patients (53.3%) in the EVT cohort, corresponding to an absolute between-cohort difference of 33.1 percentage points. Favorable functional outcome occurred in 41 of 44 patients (93.2%) and 21 of 30 patients (70.0%), respectively, with an absolute difference of 23.2 percentage points. The median 90-day mRS score was 0.0 (IQR, 0.0–1.0) in the IVT cohort and 1.0 (IQR, 1.0–2.8) in the EVT cohort (Table 7 and Figure 2C). Given the limited sample size and the small number of poor outcomes in this subgroup, these findings are presented descriptively and should be considered exploratory.

**Table 6:** Baseline characteristics of patients with MCA-M2 occlusion.

Variable	Overall (*n* = 74)	IVT (*n* = 44)	EVT (*n* = 30)	*p*
Age – year	65.0 (59.2–70.0)	65.0 (60.0–71.0)	64.5 (59.0–68.8)	0.308
Male	60 (81.1)	35 (79.5)	25 (83.3)	0.683
Current Smoking	27 (36.5)	13 (29.5)	14 (46.7)	0.133
Current Drinking	17 (23.0)	5 (11.4)	12 (40.0)	0.004
Past medical history				
Hypertension	42 (56.8)	29 (65.9)	13 (43.3)	0.054
Diabetes	12 (16.2)	8 (18.2)	4 (13.3)	0.579
Coronary heart disease	4 (5.4)	3 (6.8)	1 (3.3)	0.515
Atrial fibrillation	7 (9.5)	4 (9.1)	3 (10.0)	0.896
Ischemic stroke	26 (35.1)	16 (36.4)	10 (33.3)	0.789
Baseline NIHSS	3.0 (2.0–5.0)	2.5 (2.0–4.0)	4.5 (2.0–5.0)	0.012
SBP at admission	143.5 (132.0–159.5)	144.0 (132.0–156.0)	140.5 (126.2–166.0)	0.834
DBP at admission	82.0 (75.1–93.0)	82.0 (75.9–89.5)	85.0 (75.0–94.0)	0.843
Prestroke mRS	0.0 (0.0–0.0)	0.0 (0.0–0.0)	0.0 (0.0–0.0)	0.092
Presumed stroke cause^a^				0.215
Large artery atherosclerosis	68 (91.9)	42 (95.5)	26 (86.7)	
Cardioembolism	6 (8.1)	2 (4.5)	4 (13.3)	

Data were shown as *N* (%) or median (IQR). NIHSS, National Institutes of Health Stroke Scale; SBP, systolic blood pressure; DBP, diastolic blood pressure; IVT, intravenous thrombolysis; EVT, endovascular treatment; mRS, modified Rankin Scale; MCA-M2, M2 segment of middle cerebral artery MCA. ^a^The presumed stroke cause was classified according to the Trial of Org 10,172 in Acute Stroke Treatment (TOAST) classification system.

**Table 7:** Efficacy and safety outcomes of patients with MCA-M2 occlusion.

Outcome	IVT (*n* = 44)	EVT (*n* = 30)	*p*-value^*^
Excellent functional outcome	38 (86.4)	16 (53.3)	0.005
Favorable functional outcome	41 (93.2)	21 (70.0)	0.027
mRS distribution at 90 days^a^	0.0 (0.0–1.0)	1.0 (0.0–2.8)	0.003
Early neurological deterioration	0 (0.0)	7 (23.3)	0.003
Any intracranial hemorrhage	1 (2.3)	6 (20.0)	0.016
Symptomatic intracranial hemorrhage	0 (0.0)	4 (13.3)	0.024

Data were shown as *N* (%) or median (IQR). mRS, modified Rankin Scale; MCA-M2, M2 segment of middle cerebral artery. ^a^mRS scores range from 0 to 6: 0 = no symptoms; 1 = symptoms without clinically significant disability; 2 = slight disability; 3 = moderate disability; 4 = moderately severe disability; 5 = severe disability; and 6 = death. *p-values were obtained using unadjusted comparisons (*χ*^2^ test, Fisher’s exact test, or Mann–Whitney *U* test, as appropriate). Because of the limited sample size and sparse outcome events, adjusted regression analyses were not performed for this subgroup, and the findings should be interpreted as exploratory.

## Discussion

In this study, no clear difference in overall functional outcomes was observed between patients receiving EVT in the DETECT2-China cohort and those receiving IVT in the EAST cohort among patients with minor stroke with anterior circulation LVO. However, the observed associations differed according to occlusion site. Among patients with proximal vessel occlusion (ICA or MCA-M1), the observed point estimate for excellent functional outcome was numerically higher in the EVT cohort than in the IVT cohort. However, because treatment assignment was inherently linked to source cohort, this observation should not be interpreted as evidence of treatment benefit. Additionally, EVT was linked to numerically higher rates of sICH and END compared to IVT, although these differences were not statistically significant.

The direction of the observed associations in patients with proximal occlusion supports the need for dedicated randomized trials to determine the optimal reperfusion strategy in this subgroup. These findings primarily highlight heterogeneity in observed outcomes across occlusion locations and support the importance of considering occlusion site when designing future comparative studies and clinical trials (26–29). Previous studies indicate that proximal occlusions are often associated with larger ischemic territory and higher risk of early neurological deterioration, and are less likely to achieve favorable functional outcomes without successful recanalization (8, 26, 30–33). These characteristics may partly explain why outcomes in the EVT cohort appeared more favorable among patients with ICA or MCA-M1 occlusion. However, this interpretation remains hypothesis-generating. Nevertheless, because treatment assignment was inherently determined by source cohort: IVT in the EAST cohort and EVT in the DETECT2-China cohort, these findings should not be interpreted as evidence of treatment benefit or harm. Differences between the cohorts may reflect not only the reperfusion strategy but also variations in study design, enrollment period, participating centers, imaging practices, procedural selection, and other measured or unmeasured patient characteristics. In contrast, EVT for distal occlusions is technically more challenging and may carry increased risks of procedure-related complications, such as vessel perforation, distal embolization, or hemorrhagic transformation (34, 35), which may be especially concerning in patients with low baseline NIHSS scores (26, 27).

Our results align with previous studies that have expressed caution regarding the routine use of EVT in patients with minor stroke (26–28). In particular, several subgroup analyses have indicated potentially harmful effects of EVT in patients with M2 occlusions and low NIHSS scores, especially in the absence of significant perfusion mismatch or early infarct progression (28). These exploratory findings suggest that the decision to pursue EVT should not rely solely on the presence of LVO, but should instead incorporate a comprehensive evaluation that includes occlusion location, infarct core volume, collateral status, and clinical-imaging mismatch. Another important difference across published studies is the reperfusion strategy being evaluated. Several previous studies compared bridging therapy with IVT alone in IVT-eligible patients, whereas others evaluated EVT (with or without prior IVT) against medical management (26, 36). In our study, only 16 of 132 patients (12.1%) in the EVT cohort received bridging therapy, while the majority underwent direct EVT. Therefore, our findings primarily reflect differences between a predominantly direct EVT cohort and an IVT cohort rather than the clinically important comparison between bridging therapy and IVT alone. This distinction should be considered when comparing our results with those of previous studies.

From a clinical standpoint, our study highlights the need for further evaluation of a selective and individualized approach to reperfusion therapy in minor stroke patients. Treatment decisions should integrate vascular and perfusion imaging to carefully weigh potential benefits against procedural risks, particularly for M2 occlusions. However, given the observational comparison between two distinct source cohorts, the present study cannot establish whether one reperfusion strategy is preferable to the other. Treatment decisions should therefore continue to be based on multidisciplinary clinical judgment and the totality of available imaging and patient-specific information.

Several limitations of our study warrant acknowledgment. First, although this pooled cohort incorporates data from an RCT and multiple high-volume centers, the analysis was conducted retrospectively with a relatively small sample size. This may introduce confounding biases, limit statistical power, and restrict the generalizability of our findings. More importantly, treatment assignment was completely aligned with source cohort: patients receiving IVT were derived from the EAST cohort, whereas those receiving EVT were derived from the DETECT2-China cohort. Consequently, treatment strategy was inseparable from differences in study period, center characteristics, eligibility criteria, imaging protocols, procedural practices, and patient selection. Although statistical adjustment was performed, residual and unmeasured confounding likely remained, and the significant interaction *p* value should not be regarded as evidence of true treatment-effect modification, and the subgroup findings are exploratory and hypothesis-generating only. Second, detailed perfusion imaging was not uniformly available, preventing assessment of the ischemic penumbra and dynamic infarct characteristics. Third, we were unable to evaluate collateral status or its association with EVT outcomes. Additionally, specific presenting symptoms were not documented for all patients, precluding symptom- or syndrome-based analyses. Fourth, since only IVT-eligible patients were included, our results may not extend to those ineligible for IVT, especially patients presenting beyond 4.5 h from symptom onset. Ultimately, definitive guidance regarding the optimal reperfusion strategy for minor stroke with LVO will require dedicated randomized controlled trials, such as the ongoing ENDOLOW (NCT04167527) and MOSTE (NCT03796468) studies. These trials are expected to provide more definitive evidence regarding patient selection and the relative benefits and risks of EVT and medical treatment in this population.

## Conclusion

In this exploratory analysis, the association between reperfusion strategy and functional outcome differed according to occlusion site. Patients with proximal occlusions (ICA or MCA-M1) in the EVT cohort had numerically better outcomes, whereas those with M2 occlusions in the EVT cohort had worse outcomes compared with the IVT cohort. However, these findings are hypothesis-generating and require prospective validation in ongoing randomized trials.

## Data Availability

The original contributions presented in the study are included in the article/Supplementary material, further inquiries can be directed to the corresponding author.
