# Supplementary material for: Association of endovascular treatment versus intravenous thrombolysis in mild stroke across occlusion subgroups
Source: Front Neurol. 2026 Oct 14;17:1878069. doi: 10.3389/fneur.2026.1878069 (PMC13713266; doi:10.3389/fneur.2026.1878069)
Supplement: Supplementary file 1 [file Table_1.doc]

eTable 1. Baseline Characteristics of patients with proximal vessel occlusion (ICA or MCA-M1)

| Variable | Overall (n=163) | IVT (n=61) | EVT (n=102) | p |
| --- | --- | --- | --- | --- |
| Age - year | 67.0 (61.0 - 71.0) | 68.0 (61.0 - 74.0) | 67.0 (61.0 - 70.8) | 0.123 |
| Male | 131 (80.4) | 50 (82.0) | 81 (79.4) | 0.691 |
| Current Smoking | 79 (48.5) | 31 (50.8) | 48 (47.1) | 0.642 |
| Current Drinking | 63 (38.7) | 20 (32.8) | 43 (42.2) | 0.234 |
| Past medical history |  |  |  |  |
| Hypertension | 100 (61.3) | 35 (57.4) | 65 (63.7) | 0.421 |
| Diabetes | 45 (27.6) | 17 (27.9) | 28 (27.5) | 0.954 |
| Coronary heart disease | 7 (4.3) | 5 (8.2) | 2 (2.0) | 0.057 |
| Atrial fibrillation | 6 (3.7) | 2 (3.3) | 4 (3.9) | 0.833 |
| Ischemic stroke | 27 (16.6) | 10 (16.4) | 17 (16.7) | 0.964 |
| Baseline NIHSS | 4.0 (3.0 - 5.0) | 4.0 (3.0 - 5.0) | 4.0 (3.0 - 5.0) | 0.185 |
| SBP at admission | 144.0 (129.2 - 158.4) | 143.0 (130.0 - 158.0) | 144.0 (128.0 - 159.0) | 0.990 |
| DBP at admission | 82.0 (75.0 - 90.0) | 80.0 (75.0 - 90.0) | 82.0 (75.0 - 90.0) | 0.765 |
| Prestroke mRS | 0.0 (0.0 - 0.0) | 0.0 (0.0 - 0.0) | 0.0 (0.0 - 0.0) | 0.564 |
| Presumed stroke cause^a^ | |  |  | 0.309 |
| Large artery atherosclerosis | 149 (91.4) | 54 (88.5) | 95 (93.1) |  |
| Cardioembolism | 14 (8.6) | 7 (11.5) | 7 (6.9) |  |

Data were shown as N (%) or median (IQR). Abbreviations: ICA, internal carotid artery; MCA-M1, M1 segment of middle cerebral artery MCA; NIHSS, National Institutes of Health Stroke Scale; SBP - systolic blood pressure; DBP - diastolic blood pressure; IVT, intravenous thrombolysis; EVT, endovascular treatment; mRS, modified Rankin Scale.

a The presumed stroke cause was classified according to the Trial of Org 10172 in Acute Stroke Treatment (TOAST) classification system.

eTable 2. Baseline Characteristics of patients with MCA-M2 occlusion

|  | Overall (n=74) | IVT (n=44) | EVT (n=30) | p |
| --- | --- | --- | --- | --- |
| Age - year | 65.0 (59.2 - 70.0) | 65.0 (60.0 - 71.0) | 64.5 (59.0 - 68.8) | 0.308 |
| Male | 60 (81.1) | 35 (79.5) | 25 (83.3) | 0.683 |
| Current Smoking | 27 (36.5) | 13 (29.5) | 14 (46.7) | 0.133 |
| Current Drinking | 17 (23.0) | 5 (11.4) | 12 (40.0) | 0.004 |
| Past medical history |  |  |  |  |
| Hypertension | 42 (56.8) | 29 (65.9) | 13 (43.3) | 0.054 |
| Diabetes | 12 (16.2) | 8 (18.2) | 4 (13.3) | 0.579 |
| Coronary heart disease | 4 (5.4) | 3 (6.8) | 1 (3.3) | 0.515 |
| Atrial fibrillation | 7 (9.5) | 4 (9.1) | 3 (10.0) | 0.896 |
| Ischemic stroke | 26 (35.1) | 16 (36.4) | 10 (33.3) | 0.789 |
| Baseline NIHSS | 3.0 (2.0 - 5.0) | 2.5 (2.0 - 4.0) | 4.5 (2.0 - 5.0) | 0.012 |
| SBP at admission | 143.5 (132.0 - 159.5) | 144.0 (132.0 - 156.0) | 140.5 (126.2 - 166.0) | 0.834 |
| DBP at admission | 82.0 (75.1 - 93.0) | 82.0 (75.9 - 89.5) | 85.0 (75.0 - 94.0) | 0.843 |
| Prestroke mRS | 0.0 (0.0 - 0.0) | 0.0 (0.0 - 0.0) | 0.0 (0.0 - 0.0) | 0.092 |
| Presumed stroke cause^a^ | |  |  | 0.092 |
| Large artery atherosclerosis | 68 (91.9) | 42 (95.5) | 26 (86.7) |  |
| Cardioembolism | 6 (8.1) | 2 (4.5) | 4 (13.3) |  |

Data were shown as N (%) or median (IQR). Abbreviations: NIHSS, National Institutes of Health Stroke Scale; SBP, systolic blood pressure; DBP, diastolic blood pressure; IVT, intravenous thrombolysis; EVT, endovascular treatment; mRS, modified Rankin Scale; MCA-M2, M2 segment of middle cerebral artery MCA.

a The presumed stroke cause was classified according to the Trial of Org 10172 in Acute Stroke Treatment (TOAST) classification system.
